# Cytogenetic studies on populations of *Camponotus rufipes* (Fabricius, 1775) and *Camponotus renggeri* Emery, 1894 (Formicidae: Formicinae)

**DOI:** 10.1371/journal.pone.0177702

**Published:** 2017-05-16

**Authors:** Hilton Jeferson Alves Cardoso de Aguiar, Luísa Antônia Campos Barros, Danúbia Rodrigues Alves, Cléa dos Santos Ferreira Mariano, Jacques Hubert Charles Delabie, Silvia das Graças Pompolo

**Affiliations:** 1 Programa de Pós-graduação em Genética e Melhoramento, Universidade Federal de Viçosa, Viçosa, Minas Gerais, Brazil; 2 Universidade Federal do Amapá, Campus Binacional, Oiapoque, Amapa, Brazil; 3 Laboratório de Mirmecologia, CEPEC/CEPLAC, Itabuna, Bahia, Brazil; 4 Departamento de Ciências Biológicas, Universidade Estadual de Santa Cruz, Ilhéus, Bahia, Brazil; 5 Departamento de Ciências Agrárias e Ambientais, Universidade Estadual de Santa Cruz, Ilhéus, Bahia, Brazil; Chinese Academy of Agricultural Sciences Cotton Research Institute, CHINA

## Abstract

Two valid ant species, *Camponotus rufipes* and *Camponotus renggeri*, have recently been the subject of a broad discussion with reference to taxa synonymization. Both species are quite common among the Neotropical myrmecofauna and share some unique traits, such as the shape of the scape and the pilosity patterns of the tibiae and scapes. A single morphological trait can help distinguish these species; however, only a combination of different approaches can enlighten our view of the complex phylogenetic relationships prevailing in the different populations of these two taxa. Therefore, focusing on the taxonomic issues concerning these two species, a cytogenetic survey including 10 populations of *C*. *rufipes* and two populations of *C*. *renggeri* was performed. In order to better understand the extent of the relationship between *C*. *rufipes* and *C*. *renggeri*, two common Neotropical *Camponotus* species, *C*. *atriceps* and *C*. *cingulatus* were taken as outgroups. All four species of *Camponotus* that were studied had 2n = 40 chromosomes (4sm+34st+2t); however, the abundance of chromosome rearrangements observed, combined with several chromosome markers, suggest that *C*. *rufipes* and *C*. *renggeri* are two good distinct species although closely related. The already reported chromosome translocation 2n = 39 (1m+4sm+32st+2t) for *C*. *rufipes* has been found in different populations as in the unprecedented chromosome inversions found both in *C*. *rufipes* and in *C*. *renggeri* populations. Within the *C*. *renggeri* chromosome inversions, both the heterozygous state 2n = 40 (1m+3sm+34st+2t) and the homozygous state, 2n = 40 (2m+2sm+34st+2t) were identified. However, only heterozygous specimens for chromosome inversions were found among *C*. *rufipes*, with karyotype configurations distinct from those found in *C*. *renggeri*, with 2n = 40 (1m+4sm+34st+2t). None of the populations studied showed signs of mosaic individuals. With respect to rDNA clusters, the 18S rDNA seemed to be more restricted inside the genome, as *C*. *renggeri* showed four 18S rDNA clusters, whereas, *C*. *rufipes*, *C*. *atriceps*, and *C*. *cingulatus* showed only two clusters. The chromosome locations of the 5S rDNA clusters were pointed for the first time in Formicidae, and showed itself to be more widely spread over the genome. By combining different chromosome banding approaches it was possible to demonstrate the crucial importance that chromosome inversions played on the karyotype evolution within these ants. The results also showed that chromosome translocations might be a consequence of the chromatin dynamic condition observed among *Camponotus* species. The homozygosis condition found in a *C*. *renggeri* from a Brazilian savanna population for chromosome inversions and the contrasting heterozygous condition for a different kind of chromosome inversion in *C*. *rufipes* from the Brazilian coastal rainforest, opens the window for a chromosome race hypothesis within the group *C*. *renggeri* and *C*. *rufipes*. The wide distribution, rich ecological interactions, genetic diversity, and morphological variability among *C*. *renggeri* and *C*. *rufipes* justify questioning of the actual taxonomic status of these species. The answer of this puzzle is clear when observing the number of 18S rDNA clusters of these ants, as *C*. *rufipes* has only two clusters whereas *C*. *renggeri* has four.

## Introduction

There are many species of ants that form complexes of species and it is not rare to find several morphotypes (formerly called varieties) within many groups of taxa in a similar context as that discussed by Mayr [1]. It is important then, to distinguish sister species from ecologically or geographically differentiated morphotypes. According to the Biological Species Concept, two “good” species must have well-defined prezygotic or postzygotic barriers. This condition is not verified between morphotypes, races, varieties, or whatever the terminology used [2]. In keeping with this idea, Bickford et al. [3] defined a cryptic species as a group of two or more morphologically similar species hidden under one single nominal taxon. These authors do not seem to agree with the possibility of hybridism, as cryptic species are “good” species, and the level of morphological relationship would not reflect genetic distinctness. Therefore, the term species complex may be a little slippery because one should first decide if the argument is related to the topic of cryptic species or another kind of individual grouping, based on morphology. In ants the utilization of the terms varieties and races are uncommon [4]–although largely used in other groups. They are deeply studied under the context of population, some of them involving cytogenetic studies [5].

At present, *Camponotus* Mayr 1861 is the ant genus with the highest number of described species and subspecies, that is, 1,099 described taxa inserted in 43 subgenera [6]. Taxonomic relationships between subgenera and species complexes of Neotropical *Camponotus* have been intensively discussed and reviewed by Mackay [7]. Many species of this genus are widely distributed and are found in different biomes [8]. Following data accumulation on the *Camponotus* species, several discussions involving taxonomic implications became popular, such as, species synonymization [7, 8], or the occurrence of cryptic species complexes [9]. Although *Camponotus* is considered one of the most prevalent genera [10] with vast ecological diversity, there is still much to discover about the morphological variability within the valid *Camponotus* taxa, mainly relating to those with wide geographic distributions. Following this thought, the synonymization of *Camponotus rufipes* (Fabricius 1775) and *C*. *renggeri* Emery 1894, suggested by Mackay [7], gains attention because both species are abundant in several regions of South America and usually have highly contrasting color patterns. In dealing with the synonymization problem, Mackay [7] has the following to state about *C*. *rufipes*: “This species can be distinguished from others as the scape is flattened at the base (majors and females), the scape and tibiae are covered with erect hairs, the majority of the ant is dark (nearly black) with orange legs, and all surfaces are densely punctate. No other New World ant has this combination of characters. *Camponotus renggeri* is obviously a synonym [of *C*. *rufipes*].”

The *Camponotus* genus is the richest ant group and is possibly not monophyletic [11], with the phylogenetic relationship of its species based on morphological traits [6]. Several efforts have been made to organize the *Camponotus* subgenera into natural groups, but it seems that something is always out of place. The Neotropical subgenus *Myrmothrix*, which includes *C*. *rufipes*, *C*. *renggeri*, *C*. *atriceps*, and *C*. *cingulatus* is considered by Hashmi [8] to be monophyletic, by using morphological traits. However, Brady et al. [11], through COI mitochondrial DNA sequences presented a paraphyly possibility between *Myrmothrix* and *Tanaemyrmex*. Lastly, Mackay [7] suggested a new phylogenetic organization for *Camponotus* species, whereas the group (*Tanaemyrmex* + *Myrmothrix*) would be subdivided into natural subgroups, initially considered species complexes. Most of the *Myrmothrix* species, such as, *C*. *rufipes*, *C*. *renggeri*, and *C*. *atriceps* would belong to the *Atriceps* species complex, while *C*. *cingulatus* (a *Myrmothrix* species) would be part of the *Picipes* species complex alongside different species of *Tanaemyrmex* subgenus. Several taxonomic suggestions proposed by Mackay [7] are not well supported by the myrmecologists, including the synonymization of *C*. *rufipes* and *C*. *renggeri* [12]. These two species are commonly sympatric and both share several morphological and behavioral traits. So far, the dull/shiny aspect of the body and the color of the coxae are the main morphological traits that allow distinguishing of the two.

The recent advances from the so-called “integrative taxonomy” represent a promise of solving some issues by combining morphology with different fields [13, 14]. Through cytogenetics, it is possible to investigate not only the amount of genetic variability [15, 16], but also the speciation process itself [5, 17], by finding possible postzygotic barriers [18–20]. Therefore, this field of genetics may be an important tool for better understanding both the complex phylogenetic relationships and the mechanisms involved, with the creation of high levels of variability present within different ant species.

The cytogenetic information available for more than 750 ant morphospecies [21] help taxonomic discussions, such as those involving cryptic species [22–24]. Only 4% of the valid *Camponotus* species have any kind of cytogenetic information available [21] and only recently *C*. *renggeri* has been described by cytogenetic means [25] through chromosome morphology and number. On the other hand, its presupposed sister species, *C*. *rufipes* accounts with two distantly related populations studied: one from Uruguay [26] and the second from southeastern Brazil where a characteristic chromosome polymorphism has been observed [27].

*Camponotus rufipes* and *C*. *renggeri* sympatrically inhabit some areas from Cerrado (South American savannas) and Mata Atlântica (South American Atlantic coastal rainforests). The chromosome dynamics of these two taxa must be better investigated, to clarify several questions pertinent to recent taxonomic discussions about the boundaries of these species. Therefore, the present study intends to compare the chromosome configuration of different Brazilian populations of *C*. *rufipes* and *C*. *renggeri*, picking up pieces that may help to solve the complex puzzle which is the real taxonomic status of these species. The possibility of synonymization was investigated by observing the peculiarities of distinct populations from contrasting South American environments, such as Cerrado, Amazon rainforest, and Mata Atlântica (including the sandy open vegetation along the Brazilian coastline), and also the karyotypes of the closely related *Camponotus* species (subgenus *Myrmothrix*, *sensu* Hashmi [8]): *Camponotus atriceps* (Fabricius, 1804), and *Camponotus cingulatus* Mayr, 1862.

## Materials and methods

### Field work, specimen collecting and nest maintenance

The sampling of specimens was carried out in several distinct Brazilian environments, such as rainforests (Amazon and Mata Atlântica), and Savanna between 2011 and 2015 (Table 1, S1 Table). Two other *Camponotus* species were also collected: *C*. *atriceps* and *C*. *cingulatus*. All four species belong to the *Myrmothrix* subgenus [8]. In this study, *C*. *atriceps* and *C*. *cingulatus* species were considered external to the clade (*C*. *renggeri* + *C*. *rufipes*). Both *C*. *rufipes* and *C*. *renggeri* are widely distributed over South America, and therefore, the samplings were carried out in different localities allowing further environmental comparisons. As this study has qualitative purposes, only the presence of chromosome variations within these populations was observed.

**Table 1:** Cytogenetic data of *Camponotus* (*Myrmothrix*). Information connecting species; sample sites; geographic coordinates; biome and diploid chromosome number. Karyotype information: W (wild, 2n = 40), I (inversion, 2n = 40), T (translocation, 2n = 39). Cytogenetic techniques information: BC (C-banding), FL (fluorochromes CMA_3_/DAPI), NOR (NOR banding), FISH (Fluorescence *In Situ* Hybridization). Locality details: U—Uruguay; Brazillian States: MG—Minas Gerais, RJ—Rio de Janeiro, MT—Mato Grosso, AP—Amapá, PR—Paraná.

Species	Locality (coordinates)	Biome	2n (n)	Karyotype	Techniques	Reference
*C*. *atriceps*	Lavras—MG (21°13’S, 54°55’W)	Cerrado	40	W	BC, FL	Present study
*C*. *cingulatus*	Rio de Janeiro—RJ (23°00’S, 43°22’W)	Mata Atlântica	40; (20)	W	BC, FL	Present study
*C*. *cingulatus*	Viçosa—MG (20°45’S, 42°51’W)	Mata Atlântica	40	W	BC, FL, FISH	Present study
*C*. *renggeri*	Nova Mutum—MT (13°49’S, 56°05W)	Cerrado	40	W, I, I*	BC, FL, FISH	Present study
*C*. *renggeri*	Macapá—AP (0°00’S, 51°05’W)	Amazônia	40; (20)	W	BC, FL, FISH	Present study
*C*. *rufipes*	U: Piriapolis-Maldonado (34°51’S, 55°17’W)	Pampas	40	W	–	Goñi et al. (1983)
*C*. *rufipes*	U: Punta del Este-Maldonado (34°58’S, 54°57’W)	Pampas	40; (20)	W	–	Goñi et al. (1983)
*C*. *rufipes*	Viçosa—MG (20°48’S, 42°51’W)	Mata Atlântica	40, 39; (20, 19)	W, T	BC, FL, FISH	Present study
*C*. *rufipes*	Viçosa—MG (20°48’S, 42°51’W)	Mata Atlântica	40, 39	W, T	–	Mariano et al. (2001)
*C*. *rufipes*	Ponte Nova—MG (20°21'S, 42°49'W)	Mata Atlântica	40, 39	W, T, I	BC, FL, FISH	Present study
*C*. *rufipes*	Lavras—MG (21°13’S, 444°59’W)	Cerrado	40, 39	W, T	BC, FL, FISH	Present study
*C*. *rufipes*	Ubá—MG (21°05’S, 42°55’W)	Mata Atlântica	40; (20)	W, T	BC, FL, FISH	Present study
*C*. *rufipes*	Petrópolis—RJ (22°30’S, 43°13’W)	Mata Atlântica	40	W	BC, FL	Present study
*C*. *rufipes*	Rio de Janeiro—RJ (23°00’S, 43°22’W)	Mata Atlântica	40; (20)	W	BC, FL	Present study
*C*. *rufipes*	Curitiba—PR (25°26’S, 49°014’W)	Mata Atlântica	40, 39; (20)	W, T	BC, FL, FISH	Present study
*C*. *rufipes*	Urucânia—MG (20°22’S, 42°44’W)	Mata Atlântica	40	W	FL	Present study

*—homozygous chromosome inversion

Specimen collection was authorized by the Brazilian Instituto Chico Mendes de Conservação da Biodiversidade (ICMBio) according to a special collecting permit (SISBio accession number 34567–4). Ant vouchers (workers) were deposited in the reference collection of the Laboratório de Mirmecologia, Centro de Pesquisas do Cacau (CPDC/Brazil), under the record #5724.

Parts of colonies of *Camponotus* were kept alive under laboratory conditions in the Laboratório de Citogenética de Insetos of Universidade Federal de Viçosa, Brazil. Rearing colonies allowed obtaining eggs and larvae in their initial stages of development, aiming to obtain metaphasic chromosomes.

### Chromosome preparation, karyotype analysis, banding techniques and FISH

Mitotic metaphases were obtained according to Imai et al. [28], by dissecting the cerebral ganglia or testes of the larvae (after *meconium* elimination). A total of 77 nests of *C*. *rufipes*, 28 of *C*. *renggeri*, two of *C*. *cingulatus* and two of *C*. *atriceps* were analyzed including about 15 individuals per colony (S1 Table for details). Conventional Giemsa staining was used to determine the chromosome number and morphology.

The metaphases were observed and photographed using a Olympus^®^ BX 60 microscope with a 100X objective, coupled with a Q-Color3 Olympus^®^ image capture system. Chromosomes were measured and organized using Image Pro Plus^®^ and Adobe Photoshop CS^®^software packages, respectively. Non-overlapping metaphases with similar degrees of condensation were organized by pairing the chromosomes in order of size, measuring and classifying them according to Levan et al. [29], based on the chromosome arm ratio (r) and organized as metacentric (m), submetacentric (sm), subtelocentric (st) and telocentric (t). This chromosome classification allowed distinguishing minor differences in the chromosome morphology in ants with similar karyotypes [30, 31].

The C-banding technique was performed according to Sumner [32] with minor clock adaptations to detect heterochromatin suggested by Barros et al. [33]. It was noteworthy that the heterochromatin pattern could also be observed using Giemsa staining as a result of structural heterochromatin differences [28].

Metaphases of individuals from different localities were stained with sequential fluorochromes Chromomycin A_3_ (CMA_3_) and 4’,6-diamidin-2-phenylindole (DAPI), to detect regions rich in GC and AT base pairs, respectively, according to the protocol of Schweizer [34]. The metaphases were analyzed with an epifluorescence microscope using the filters WB (450–480 nm) and WU (330–385 nm) for CMA_3_ and DAPI, respectively.

The NOR banding technique was carried out to detect Nucleolus Organizer Regions (NORs) according to Howell and Black [35]. Additionally, ribosomal gene clusters were detected by Fluorescence *In Situ* Hybridization (FISH) according to Pinkel et al. [36] with the use of 18S rDNA probe [37] and 5S rDNA [38], isolated from the bee *Melipona quinquefasciata* and the ant *C*. *rufipes*, respectively. The probes 18S and 5S rDNA were labeled by an indirect method using digoxigenin-11-dUTP (Roche Applied Science), and the signal was detected with antidigoxigenin-rhodamine (Roche Applied Science). The metaphases were analyzed with an epifluorescence microscope using WU (330–385 nm) and WG filters (510–550 nm), for DAPI and rhodamine, respectively.

## Results

**Fig 1:**
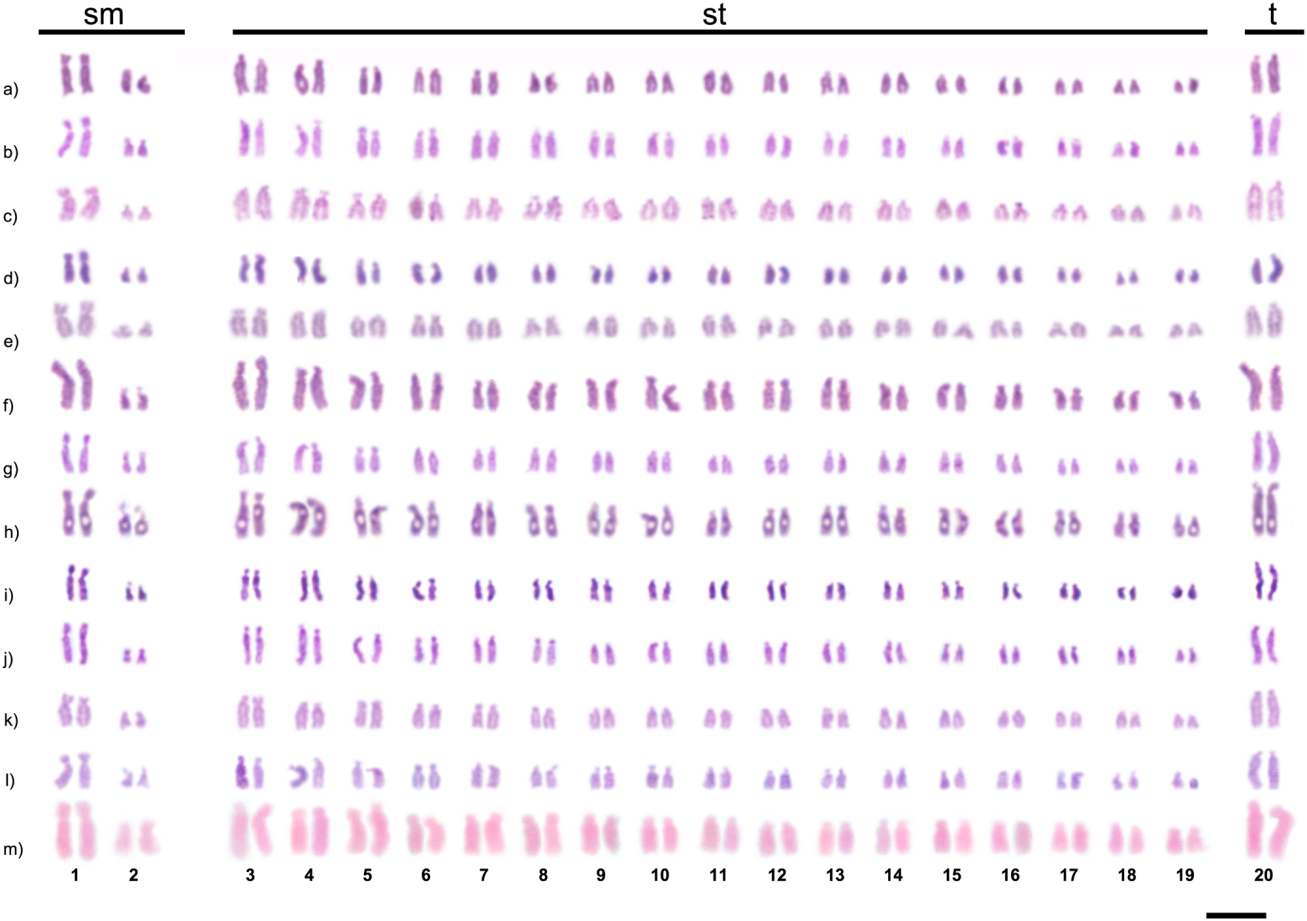
Karyotypes of *Camponotus* (*Myrmothrix*) and its localities. a) *C*. *atriceps* from Lavras; b) *C*. *cingulatus* from Viçosa; c) *C*. *cingulatus* from Rio de Janeiro; d) *C*. *renggeri* from Macapá; e) *C*. *renggeri* from Nova Mutum; f) *C*. *rufipes* from Lavras; g) *C*. *rufipes* from Viçosa; h) *C*. *rufipes* from Rio de Janeiro; i) *C*. *rufipes* from Petrópolis; j) *C*. *rufipes* from Ponte Nova; k) *C*. *rufipes* from Ubá; l) *C*. *rufipes* from Curitiba; m) *C*. *rufipes* from Urucânia. Bar = 5μm.

**Fig 2:**
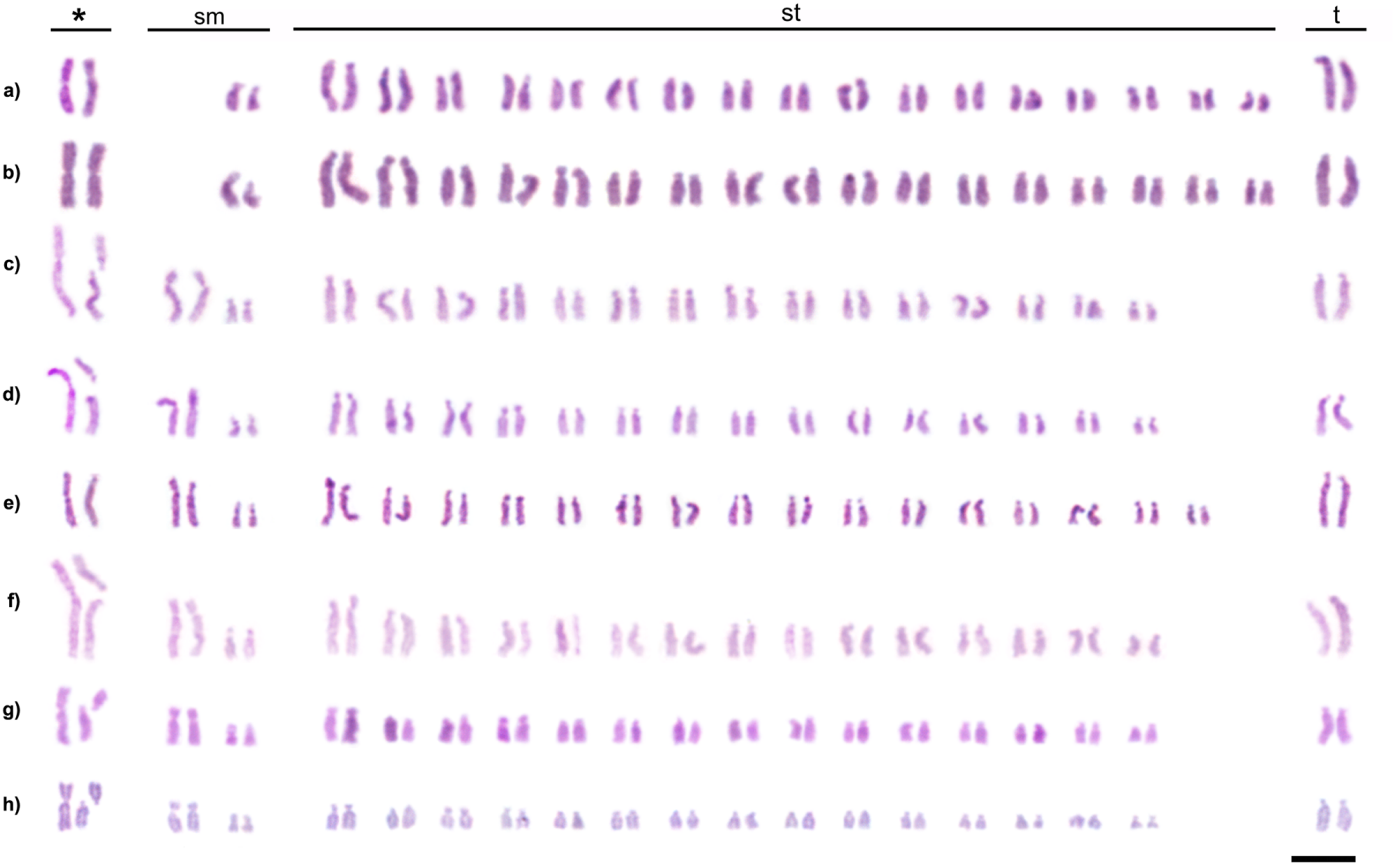
Chromosome rearrangements of *Camponotus* (*Myrmothrix*). a) *C*. *renggeri* with heterozygous chromosome inversion from Nova Mutum (2n = 40); b) *C*. *renggeri* with homozygous chromosome inversion from Nova Mutum (2n = 40); c) *C*. *rufipes* with heterozygous chromosome translocation from Lavras (2n = 39); d) *C*. *rufipes* with heterozygous chromosome translocation from Ponte Nova (2n = 39); e) *C*. *rufipes* with heterozygous chromosome inversion from Ponte Nova (2n = 40); f) *C*. *rufipes* with heterozygous chromosome translocation from Ubá (2n = 39); g) *C*. *rufipes* with heterozygous chromosome translocation from Viçosa (2n = 39); h) *C*. *rufipes* with heterozygous chromosome translocation from Curitiba (2n = 39). ✳—Rearranged chromosomes. Bar = 5μm.

All the populations studied showed a diploid chromosome number of 2n = 40, including the species *C*. *atriceps* and *C*. *cingulatus*, used as outgroup. The karyotype formula observed for the four species was 2n = 40 (4sm+34st+2t) (Fig 1). However, chromosomal variation in structure and number were seen (Fig 2, Table 1).

**Fig 3:**
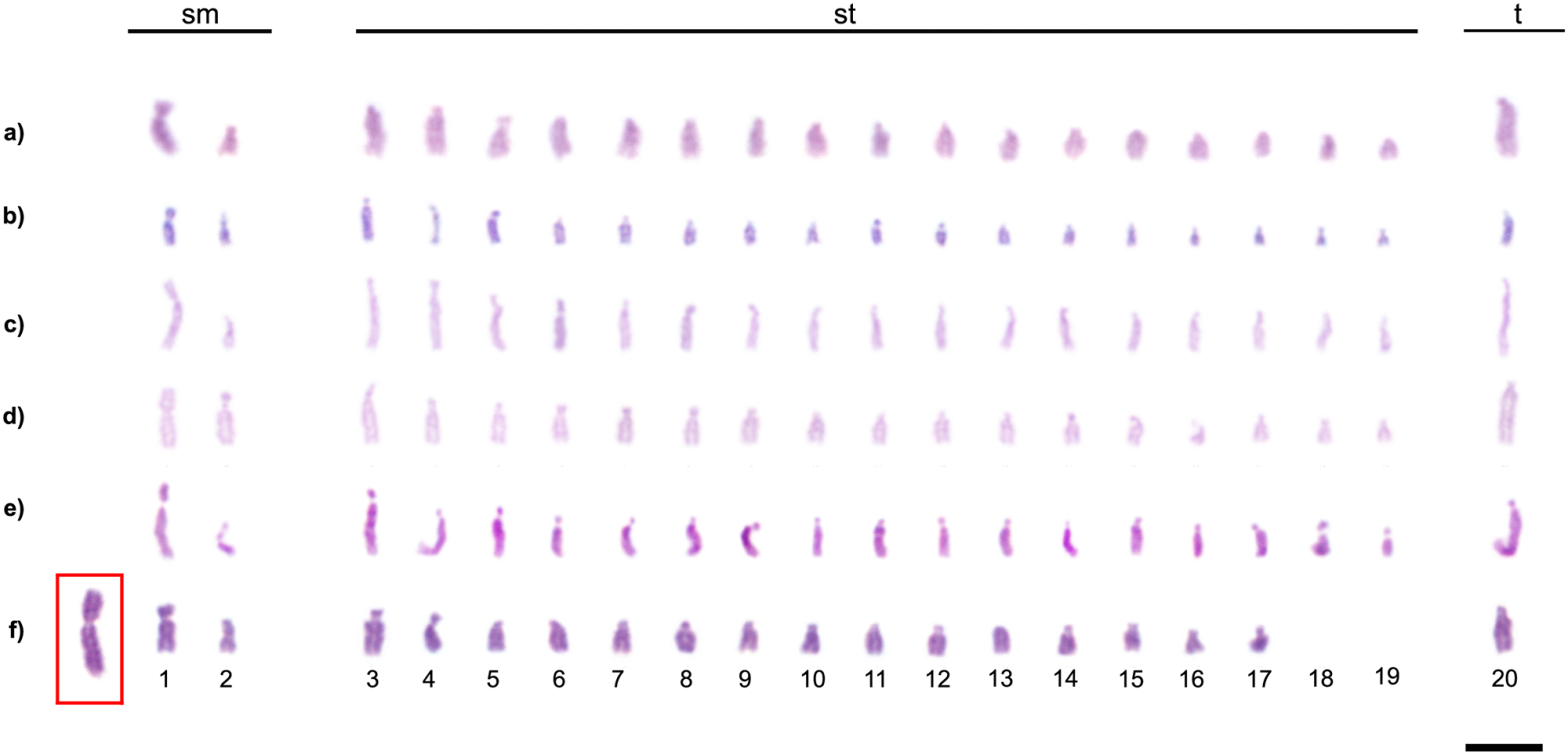
Haploid karyotypes from males of *Camponotus* (*Myrmothrix*) and its localities. a) *C*. *cingulatus* from Rio de Janeiro; b) *C*. *renggeri* from Macapá; c) *C*. *rufipes* from Rio de Janeiro; d) *C*. *rufipes* from Ubá; e) *C*. *rufipes* from Curitiba; f) *C*. *rufipes* with heterozygous translocation from Viçosa (n = 19). The box points a chromosome result of a translocation. Bar = 5μm.

In the populations of *C*. *rufipes* from the following localities: Lavras, Ponte Nova, Ubá, Viçosa, and Curitiba it was observed individuals with 2n = 39 (1m+4sm+32st+2t) (Fig 2c, 2d, 2f, 2g and 2h), a condition that characterizes a chromosome polymorphism. This will henceforth be denominated as “type I polymorphism”, hypothesized here as a Robertsonian translocation. Another kind of chromosome rearrangement was detected within *C*. *rufipes* in the population of Ponte Nova (Fig 2e) and also in *C*. *renggeri* at Nova Mutum (Fig 2a and 2b). This polymorphism was not yet described and was characterized by chromosome inversion maintaining the diploid number of wild individuals (2n = 40) changing the morphology of one of their chromosomes. It will be denominated “type II polymorphism” from now on. At Nova Mutum, nests of *C*. *renggeri* presented a heterozygous condition with 2n = 40 chromosomes (1m+3sm+34st+2t) (Fig 2a); but one colony showed the type II polymorphism in homozygous condition with 2n = 40 chromosomes (2m+2sm+34st+2t) (Fig 2b). In *C*. *rufipes*, only heterozygous individuals for this kind of rearrangement were observed with the karyotype formulae 2n = 40 (1m+4sm+33st+2t) (Fig 2e). The trait of mosaicism, the presence of two different chromosome configurations in an individual, was not found among any of the *Camponotus* individuals studied. Males of *C*. *cingulatus* (Rio de Janeiro population), *C*. *renggeri* (Macapá population) and *C*. *rufipes* (Rio de Janeiro, Ubá, Viçosa, and Curitiba populations) showed haploid karyotype n = 20 (2sm+17st+1t). Males of *C*. *rufipes* (Viçosa population) showed haploid karyotypes with n = 19 (1m+2sm+15st+1t), bearing the type I polymorphism (Fig 3). Polymorphisms involving the morphology of the chromosomes in *C*. *atriceps* and *C*. *cingulatus* were not observed. The four *Camponotus* species of the present study showed minor morphological differences between the homologous chromosomes bearing the NORs. This kind of heteromorphic condition is found, to a greater or lesser extent, in differently studied populations, as observed in Fig 1. Apart from this, an accentuated heteromorphic condition involving markings of CMA_3_ and clusters of 18S rDNA were observed in *C*. *cingulatus*.

**Fig 4:**
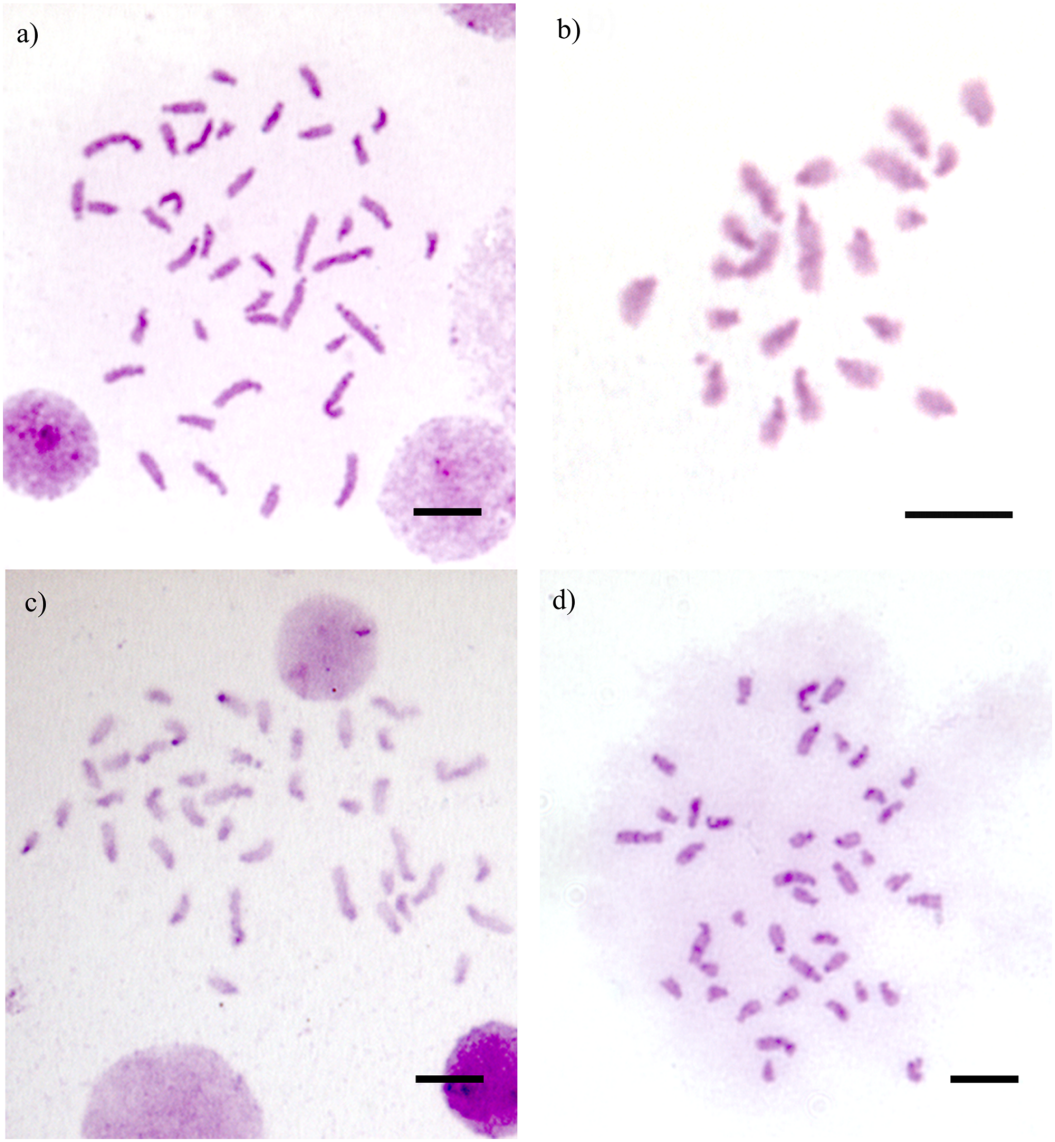
C-Banded metaphases of *Camponotus* (*Myrmothrix*). a) *C*. *atriceps* from Lavras; b) *C*. *cingulatus* from Rio de Janeiro (haploid male); c) *C*. *renggeri* from Nova Mutum; d) *C*. *rufipes* from Lavras. Bar = 5μm.

The heterochromatin pattern obtained with C-banding, and also with Giemsa staining, showed itself to be restricted to the pericentromeric region of all chromosomes, especially in their long arms (Fig 4). Some interstitial and discontinuous blocks of heterochromatin could be seen on longer chromosomes when less condensed.

**Fig 5:**
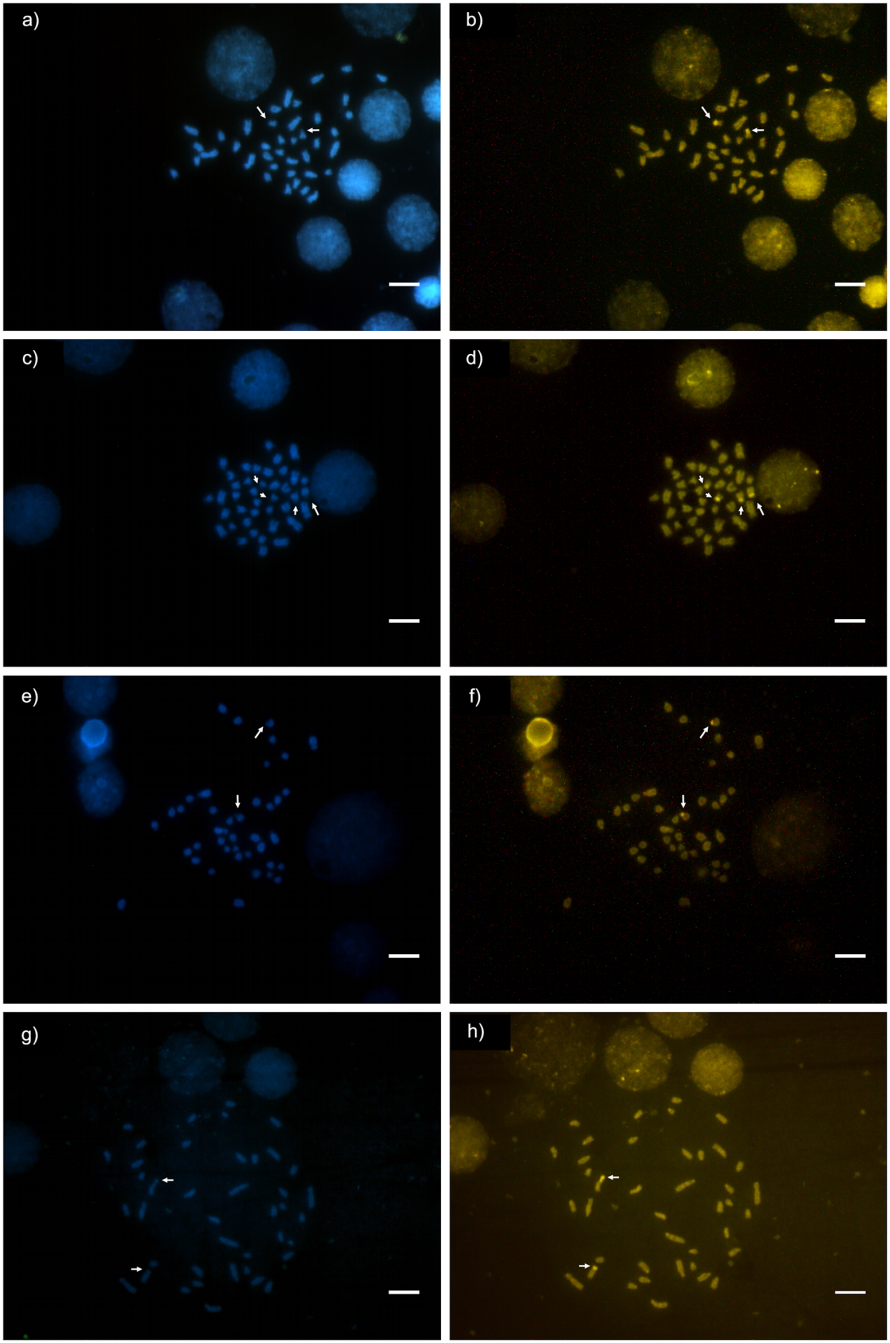
Fluorochrome treated metaphases of *Camponotus* (*Myrmothrix*). (a, b) *Camponotus rufipes*; (c, d) *C*. *renggeri*; (e, f) *C*. *atriceps*; (g, h) *C*. *cingulatus* stained with DAPI and CMA_3_ respectively. Arrows point GC-rich/AT-poor regions. Bar = 5μm.

**Fig 6:**
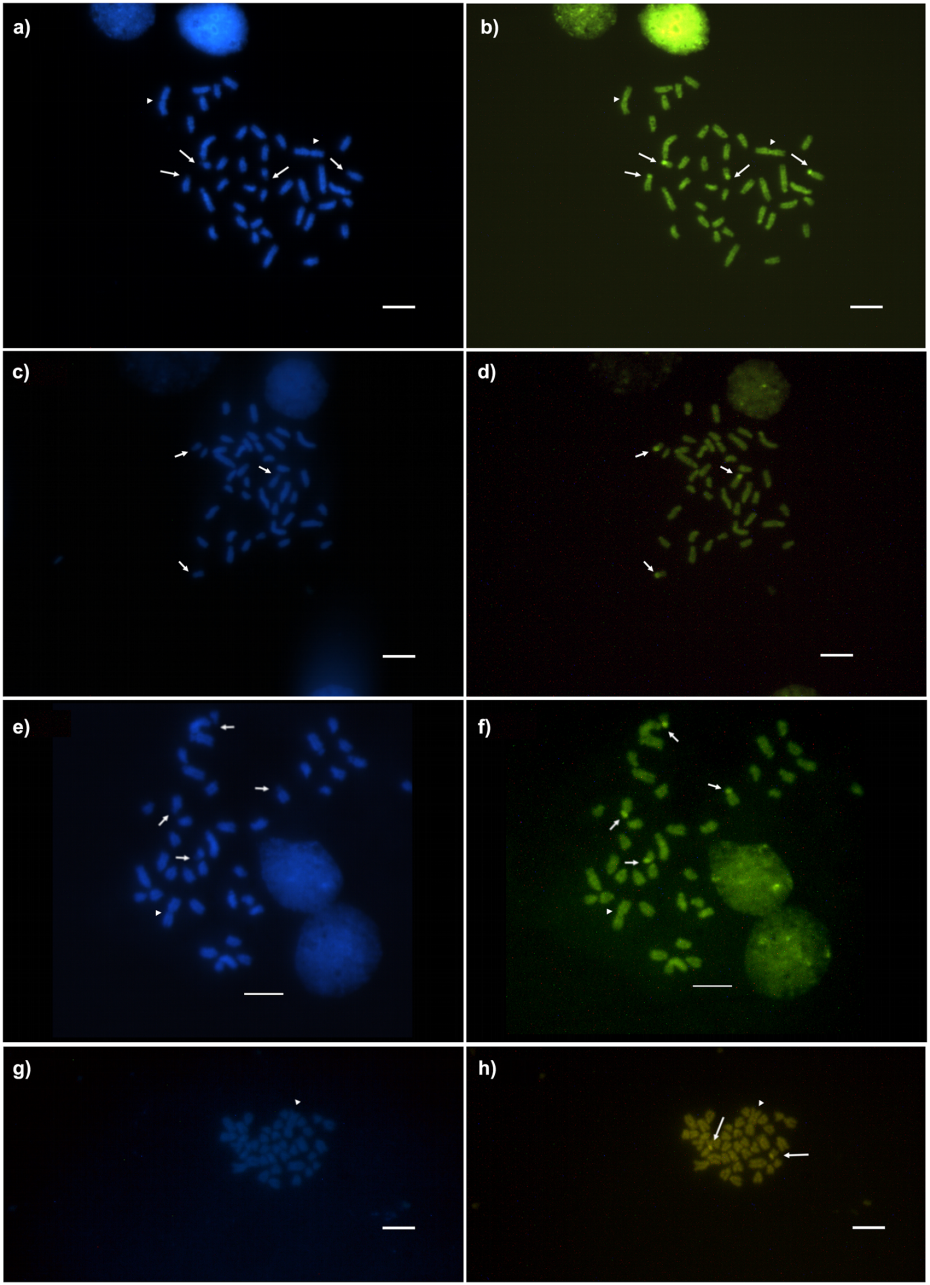
Fluorochrome treated metaphases of *Camponotus* (*Myrmothrix*) with chromosome rearrangements. All images are stained respectively with DAPI and CMA_3_. (a, b) *C*. *renggeri* with homozygous inversion (2n = 40); (c, d) *C*. *rufipes* with heterozygous translocation or hybridism (2n = 40); (e, f) *C*. *renggeri* with heterozygous inversion (2n = 40); (g, h) *C*. *rufipes* with heterozygous translocation (2n = 40). Arrows point GC-rich/AT-poor regions. Arrowheads point the rearranged chromosome. Bar = 5μm.

**Fig 7:**
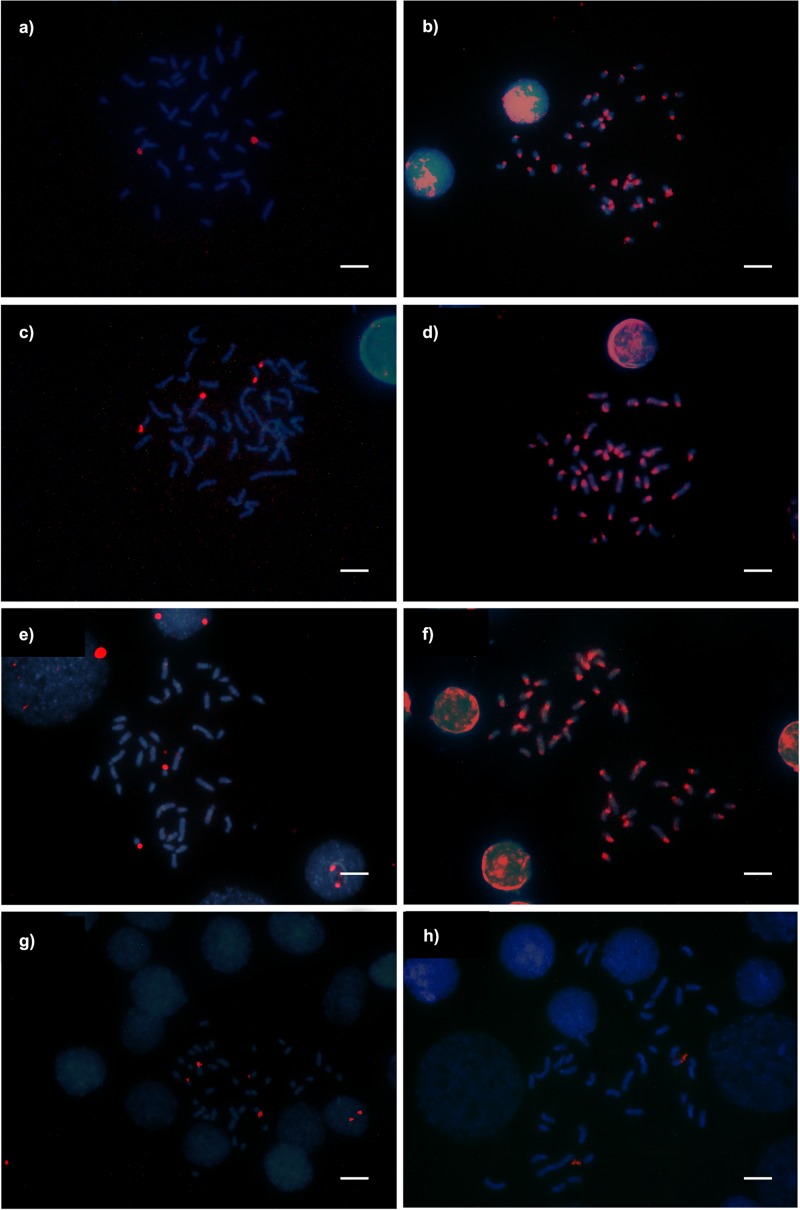
*Camponotus* (*Myrmothrix*) metaphases submitted to the FISH technique for detecting 18S and 5S rDNA clusters. (a, b) *C*. *cingulatus* from Viçosa; (c, d) *C*. *renggeri* from Nova Mutum; (e, f) *C*. *rufipes* from Viçosa showing 18S rDNA and 5S rDNA respectively; g) *C*. *renggeri* from Macapá showing 18S rDNA clusters; h) *C*. *rufipes* with heterozygous chromosome translocation (2n = 39) from Viçosa showing 18S rDNA clusters. Bar = 5μm.

Regions rich in GC base pairs using the CMA_3_ were observed in *C*. *renggeri* on the second submetacentric pair, and also on the subtelocentric pair of medium size from Macapá and Nova Mutum populations (Fig 5c). This submetacentric chromosome pair was GC-rich on the pericentromeric region of the short arm major extension, including a secondary constriction. The subtelocentric pair showed its CMA_3_^+^ region in the totality of the short arm. All the populations of *C*. *atriceps* (Fig 5a), *C*. *cingulatus* (Fig 5b), and *C*. *rufipes* (Fig 5d) had only one pair of chromosomes, the second submetacentric pair, bearing GC-rich regions. Even in the metaphases bearing rearrangements, the difference in the GC marking pattern between *C*. *rufipes* and *C*. *renggeri* was observed, indicating that they were not involved with the reported rearrangements (Fig 6). However, *C*. *rufipes* from Urucânia (MG) showed three chromosomes with blocks of rich chromatin in GC base pairs (Fig 6c and 6d). The relationship among CMA_3_^+^ regions and clusters of 18S rDNA was confirmed by the FISH technique, which showed four markings in *C*. *renggeri* (Fig 7c) and two, both in *C*. *cingulatus* and in *C*. *rufipes* (Fig 7a and 7e). A heteromorphic condition observed in *C*. *cingulatus* was confirmed by the observation of 18S rDNA genes (Fig 7a).

**Fig 8:**
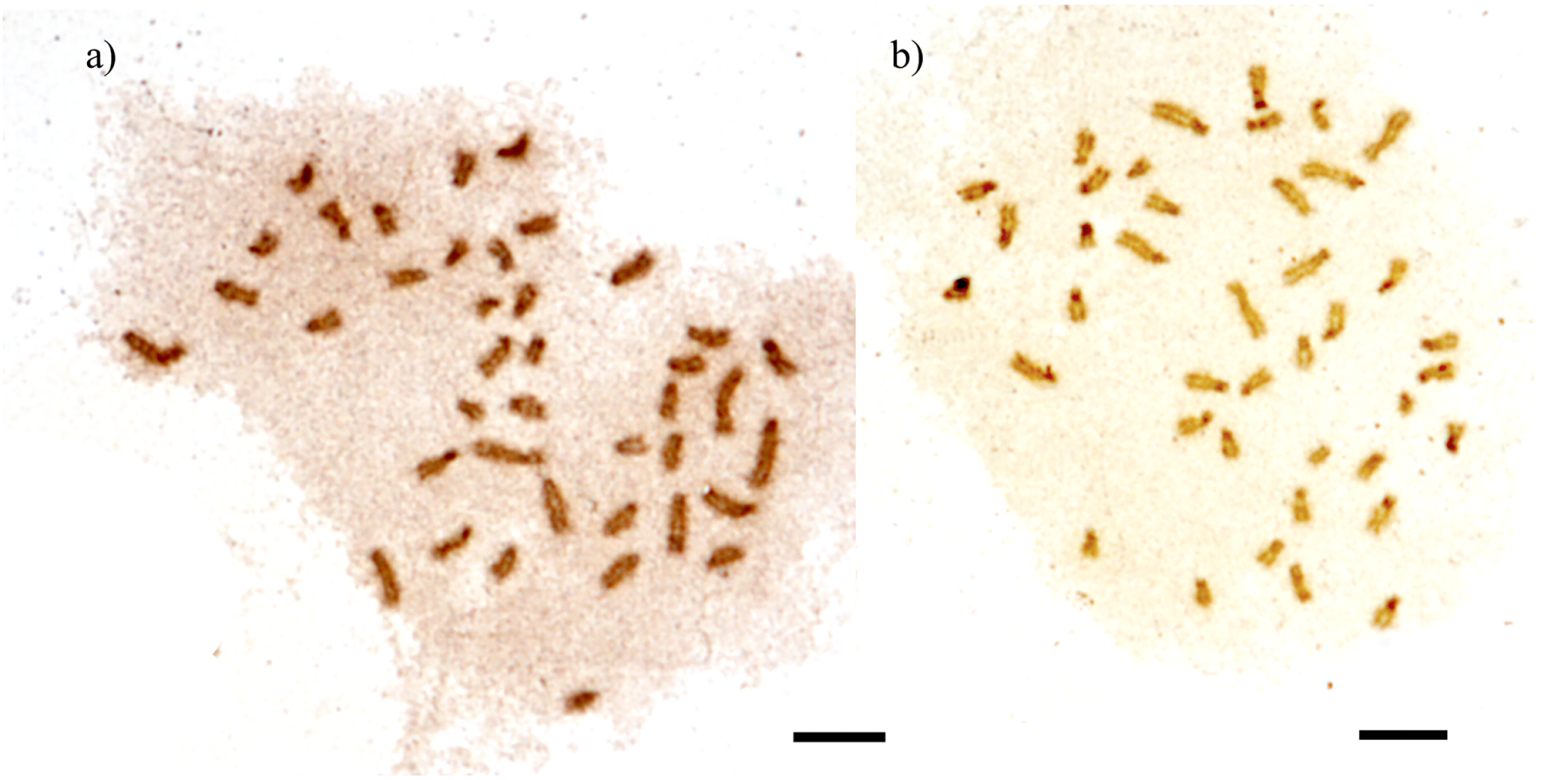
NOR banding metaphases of *Camponotus* (*Myrmothrix*). a) *C*. *renggeri* from Nova Mutum; b) *C*. *rufipes* from Lavras. Bar = 5μm.

The detection of 5S probe showed multiple marks at the pericentromeric region of all the chromosomes of *C*. *renggeri*, *C*. *rufipes* and *C*. *cingulatus* (Fig 7b, 7d and 7f). Similar results were obtained using the NOR banding technique with silver nitrate staining (Fig 8).

## Discussion

Searching the taxonomic borders of complex biological units through cytogenetic surveys has demonstrated significant advancement in better understanding species complexes [23, 39], as chromosome variations are potentially strong postzygotic barriers [40, 41]. The chromosome configuration of all the four *Camponotus* species studied here suggests a phylogenetic closeness. The novel type II polymorphism in both studied populations resulted from inversion rearrangements, but with different origins. In *C*. *renggeri*, the origin of the rearranged chromosome was from the higher submetacentric to the metacentric. In *C*. *rufipes*, the putative origin of the rearranged chromosome was from one subtelocentric to the metacentric. These mutated metacentric chromosomes were easily detected, as the four species of *Camponotus* studied here had a majority of their chromosomes with terminal centromeres.

The patterns of heterochromatin do not show measurable variations among populations or taxa. The majority of chromosomes have minor amounts of pericentromeric heterochromatin and also small blocks in the short arms of the chromosomes. Small interstitial blocks of heterochromatin dispersed within the long arms of the largest chromosomes were observed in the four *Camponous* species. As these interstitial heterochromatic blocks are not continuous in the chromosome long arms, the chromosome evolution of this group of ants may not be restricted to the fission cycle followed by tandem heterochromatin growth, such as that described by Imai et al. [42]. The uniformity of the chromosome numbers within the *Myrmothrix* subgenus clarifies the absence of chromosome fission/fusion, but chromosome inversions may have a great impact on karyotype evolution in these ants.

The combined patterns of C-banding, NOR-banding and 5S FI*S*H technique helped in the understanding of the terminal heterochromatin of the species studied. Both C-banding and NOR-banding indicated that the terminal heterochromatin regions are argentophilic. This characteristics has been reported by Sumner [43] and also recently by Cristiano et al. [44] on the Anthophoridae bee *Melitoma segmentaria* (Fabricius, 1804). However, for the first time, this trait was linked to the presence of 5S rDNA clusters. As this is the first study, to our knowledge, which has successfully detected 5S rDNA clusters in karyotypes of Formicidae, further observations will be useful in understanding this special pattern of heterochromatin.

The 18S rDNA clusters of *C*. *rufipes*, *C*. *renggeri*, and *C*. *cingulatus* have been carried out through the observation of GC-rich chromatin regions. These clusters were further confirmed by the FISH technique. The studied populations of *C*. *renggeri* have four clusters of 18S rDNA, whereas all populations of the species *C*. *rufipes* and *C*. *cingulatus* have only two clusters of this kind of 18S rDNA. Among eukaryotes, the GC-rich chromatin usually corresponds to the NORs [45], as successfully reported in other ant species, where the FI*S*H and NOR banding were performed [46–49]. The type I chromosome rearrangement is probably not an isolated event among *C*. *rufipes*, and was detected in different populations. Chromosome translocations may be connected to the origin of the additional 18S rDNA pair of the *C*. *renggeri*. This hypothesis is supported by the absence of a secondary constriction in the chromosome pair, which carries both the extra 18S rDNA clusters and the GC-rich chromatin. Among *C*. *rufipes*, *C*. *atriceps*, and *C*. *cingulatus*, only a single chromosome pair has a secondary constriction, and this same chromosome pair also carries both the 18S rDNA clusters and the GC-rich chromatin. Chromosome heteromorphic conditions connected to the NORs are not uncommon (e.g., [50, 51]).

According to the Minimum Interaction Theory (MIT) [28, 42], the evolution of chromosome morphology is directional and the selection is disruptive due to the selection against the heterozygous individuals. High numbers of chromosomes with small sizes would reduce the possibility of deleterious chromosome interactions inside the nucleus, assuming the consistency of the interphase nucleus volume, and that the chromosomes remain anchored to the nuclear membrane by their telomeres [52, 53]. These chromosome interactions produce at first heterozygous individuals. Imai [54] indicates that the growth of the heterochromatin blocks play a central role in changing the chromosome morphology, but none of the four *Camponotus* species studied have any large heterochromatin blocks at the chromosome telomeres.

It is suggested that both paracentric and pericentric inversions occur simultaneously with chromosome evolution in this group of species. According to Hoffmann et al. [55] and Hoffmann and Rieseberg [56] gene clusters that display epistatic interactions tend to remain together. Therefore, the fitness of a karyotype which undergoes chromosome inversion depends on the protection of such a group of genes from crossing-over. This seems to be a hypothesis to explain the absence of metacentric chromosomes among the species of ants from *Myrmothrix* subgenus, a chromosome configuration that allows the fission-inversion chromosome cycle to continue, as predicted by the MIT. The abundance of chromosomes with terminal centromeres may be a result of specific epistatic interactions between genes or groups of genes present in the long arms of the chromosomes. An alternative explanation could be that it is due to the centromere drive [57]. This karyotype pattern is completely different from that observed in the ant *Camponotus* (*Myrmobrachys*) *crassus* Mayr, 1862 (2n = 20) characterized by lower chromosome numbers, all of them metacentric [25, 27]. This demonstrates the rich chromosome diversity and the distinct array of evolutionary chromosome paths existing inside the *Camponotus* genus.

The chromosome rearrangements detected in the present study, translocations or inversions, are potentially able to enhance the genetic variability among the populations of these ant species. The occurrence of males with such rearrangements only increases the possibilities of chromosome exchange between populations (Fig 3f). However, these rearrangements lead to an inconvenient problem, because heterozygotes that bear these rearranged chromosomes would normally produce only 50% of balanced gametes. Some rodent species show high hybridism levels, leading to an F1 heterozygous for Robertsonian translocations. The negative effects on the fertility among them are variable but not necessarily high [58, 59]. The nests of *C*. *rufipes* and *C*. *renggeri* are large and populous and according to Ronque et al. [12], they are polygynous, which means that workers of a single colony can be the offspring of different reproductive females. Therefore, the nest would be able to survive even with heterozygous queens, producing heterozygous alates for chromosome rearrangements. However the effects on the fertility among reproductive castes of *Camponotus* species bearing rearranged chromosomes are yet unknown and should be investigated in future studies.

*Camponotus rufipes* and *C*. *renggeri* exist in sympatry in different localities over the range of their distribution, and at Urucânia, the chromosome marker CMA_3_/DAPI showed individuals with intermediary cytogenetic configurations. *C*. *rufipes* have a single pair of chromosomes marked with CMA_3_, whereas *C*. *renggeri* have two pairs marked. Intermediary individuals presented three chromosomes marked with this fluorochrome. These individuals were collected in nests with ants morphologically identified as *C*. *rufipes*. There are two hypotheses to explain this new finding: the first one suggests that the queen of the nest bears a third kind of chromosome rearrangement in a heterozygous condition, characterized by a chromosome translocation involving the rDNA 18S cluster; the second hypothesis deals with hybridization events. Unfortunately, it was not possible to obtain cytogenetic data of *C*. *renggeri* from Urucânia, which means these questions need to be discussed in further studies. Besides, Ronque et al [12] did not find data that leads to the hybridism hypothesis, and therefore, further approaches in different sympatric populations may enlighten this discussion.

Nowadays, *Camponotus* is the ant genera with the highest number of species closely followed by the Myrmicinae *Pheidole*, according to Bolton [6], and many of *Camponotus*, like the four species studied in this survey, are widely distributed over extensive geographic areas. The discussion about the synonymization of *C*. *renggeri* and *C*. *rufipes* is a result of the accumulation of morphological data on several populations. These discussions reveal the presence of “lumper” behaviors [60, 61], uncommon among myrmecologists since the emphatic study of Wilson & Brown [4]. Formicidae is considered an “ultra-diverse” group, and some may believe that the pursuit of the 20,000 valid species proposed by Hölldobler & Wilson [62] may actually represent what Mallet [63] called “taxonomic inflation”. Comprehending the many *Camponotus* taxa by organizing them in species complexes seems welcome, mainly when we focus on species with high heritable variability and large geographic distribution, such as *C*. *rufipes* and *C*. *renggeri*. The cytogenetic variability and population dynamics of these two species can be compared with those found in some well-known chromosome races of rodents, in which there is a possibility of genetic interchange, but with constraints [5], as nests with the type II rearrangement in homozygosis were found at Nova Mutum. The possibility of chromosome races within and between these taxa may also be connected to the origin of all rearrangements described in this study. This hypothesis encompasses not only the possibility of hybridization, but also deals with the role played by chromosome rearrangements in the formation of postzygotic barriers during the speciation process.

Different approaches using morphology [8], ecology, and molecular techniques [12] deal with the taxonomic problem of *C*. *rufipes* and *C*. *renggeri*, and now by observing the number of chromosomes bearing 18S rDNA clusters, it is possible to detect a discrete heritable trait, which is able to differentiate these two *Camponotus* species in different geographic locations and contrasting environments.

## Supporting information

S1 Table Collected sites, studied species and number of the colonies cytogenetically surveyed. MG—state of Minas Gerais, RJ—state of Rio de Janeiro, PR—state of Paraná, MT—state of Mato Grosso, AP—state of Amapá.
(XLSX)
